# Cochlear Implantation in Children with Autism Spectrum Disorder: A Narrative Review

**DOI:** 10.3390/healthcare14121740

**Published:** 2026-06-16

**Authors:** Irina-Maria Marinescu, Dan-Cristian Gheorghe, Alexandra Cristina Neagu, Artemis-Camelia Florescu, Andrei Borangiu, Ana-Maria Şchiau, Adina Zamfir-Chiru-Anton

**Affiliations:** 1 Department of Otolaryngology (ENT), University of Medicine and Pharmacy “Carol Davila”, 050474 Bucharest, Romania; irina-maria.marinescu@drd.umfcd.ro (I.-M.M.); dan.gheorghe@umfcd.ro (D.-C.G.); zamfiradina@yahoo.com (A.Z.-C.-A.); 2 Department of Otolaryngology (ENT), Emergency Clinical Hospital for Children “Marie S. Curie”, 75534 Bucharest, Romania; 3 Department of Otolaryngology (ENT), Emergency Clinical Hospital for Children “Grigore Alexandrescu”, 011743 Bucharest, Romania

**Keywords:** cochlear implantation, autism spectrum disorder (ASD), language acquisition, social interaction, neurodevelopmental disorders, auditory rehabilitation

## Abstract

Background/Objectives: Cochlear implantation (CI) represents a well-established intervention for the management of severe to profound sensorineural hearing loss. The co-occurrence of severe hearing loss and Autism Spectrum Disorder (ASD) presents unique diagnostic and therapeutic challenges that significantly impact post-implantation outcomes. This review aims to synthesize the current literature on cochlear implantation in children with Autism Spectrum Disorder (ASD), including diagnostic, audiological, rehabilitative, and functional outcome considerations. Methods: A structured search of PubMed and Scopus was performed for English-language articles published between January 2000 and January 2026, focusing on audiological assessment, rehabilitation challenges, multidisciplinary management, and post-implant functional outcomes in this population. Results: The findings synthesized in this review suggest that cochlear implantation in children with Autism Spectrum Disorder must be interpreted within a broader communicative-ecological framework rather than through auditory metrics alone. These findings highlight a multidimensional model of post-implant outcomes, shaped by the dynamic interplay between auditory access, social engagement, family context, and language-learning environments. Conclusions: Most children with ASD and severe-to-profound hearing loss show improvements in speech perception and production after cochlear implantation, although outcomes are highly variable. A multidisciplinary approach, through coordinated collaboration among specialists, enhances family engagement, optimizes compliance with care plans, and ultimately contributes to improved clinical and developmental outcomes. ASD should not be considered a contraindication for CI; however, careful individual assessment, realistic parental counseling, and a multidisciplinary approach availability to evaluation and rehabilitation are essential.

## 1. Introduction

A cochlear implant is a surgically implanted device for the treatment of severe to profound sensorineural hearing loss in children and adults. It works by transducing acoustic energy into an electrical signal, which is used to stimulate surviving spiral ganglion cells of the auditory nerve [1]. Deafness in pediatric age can adversely impact language acquisition, as well as educational and social-emotional development [2]. Cochlear implants have undergone substantial technological and clinical advancements, and they are now widely recognized as the standard therapeutic approach for children with severe to profound hearing loss [2]. Over the past two decades, the number of cochlear implant surgeries has increased dramatically [1].

Over the years, patient candidacy has been expanded, and the criteria for implantation continue to evolve within the paediatric population. As a result, candidacy guidelines have expanded to include both pre- and postlingually deaf children as young as 1 year of age, and also those with greater degrees of residual hearing [1]. Ongoing advances in programming methods, implant design, and atraumatic surgical techniques have confirmed the safety and effectiveness of cochlear implantation [1]. Early cochlear implantation, particularly between 12 and 18 months of age is acknowledged to optimize auditory access to speech sounds during a critical period of development, ensuring stimulation within a safe and effective listening range. In contrast, for children with severe to profound hearing loss, conventional amplification alone is often insufficient to support adequate language acquisition, highlighting the need for more advanced auditory rehabilitation strategies [2].

### 1.1. Transition to Comorbidity (ASD)

Autism spectrum disorder (ASD) is a multifaceted neurodevelopmental condition marked by impairments in social interaction, communication, and the presence of repetitive or restricted patterns of behavior. Research indicates that individuals with ASD have a higher prevalence of several co-occurring conditions compared with the general population. For example, depression, anxiety, and sleep disturbances occur more frequently among individuals with ASD [3].

Despite the widespread success of CI programs, clinical practice recognizes that certain conditions introduce significant complexities. Specifically, patients with cognitive or developmental disorders require careful consideration prior to implantation. Similarly, deaf children presenting with special clinical situations, including additional disabilities, can still be successfully treated, though this necessitates individualized candidacy evaluation and complex post-implantation rehabilitation. Within this context of complex needs, a subpopulation demanding focused research is children with Cochlear Implants and Autism Spectrum Disorder (ASD). The co-occurrence of severe hearing loss and ASD presents unique diagnostic and therapeutic challenges that significantly impact post-implantation outcomes. However, patients with cognitive or developmental disorders require careful consideration prior to implantation [4,5].

Epidemiological data indicate a steady increase in the reported prevalence of Autism Spectrum Disorder (ASD) in the pediatric population. Current epidemiological data regarding Autism Spectrum Disorder (ASD) remain limited in many regions, particularly in low- and middle-income countries. Recent global estimates suggest an overall ASD prevalence of approximately 0.77%, with higher rates consistently reported among males. However, substantial geographic variability has been observed worldwide. Lower prevalence estimates have been reported in certain regions of South-East Asia, whereas considerably higher rates have been described in Europe, the Americas, and several high-income countries, including Sweden and Australia. These regional differences are likely multifactorial and may reflect variations in study methodology, diagnostic practices, healthcare infrastructure, accessibility to specialized neurodevelopmental services, public awareness, sociocultural influences, and the potential underrecognition or underdiagnosis of ASD in resource-limited settings [4,6].

In addition to differences in diagnostic infrastructure and healthcare accessibility, environmental exposures, urbanization-related lifestyle factors, and sociocultural influences may also contribute to the regional variability observed in ASD prevalence. Several studies have suggested that urban residence may be associated with increased ASD prevalence, potentially reflecting both environmental influences and differences in community awareness, healthcare access, and identification practices [7].

Hearing impairment and Autism Spectrum Disorder frequently coexist, with ASD reported in approximately 7–9% of Deaf and Hard-of-Hearing (D/HH) children, compared with nearly 2% in the general pediatric population [8].

The relationship between ASD and peripheral hearing impairment remains incompletely understood. Although current evidence has not demonstrated a clearly increased risk of peripheral hearing loss in children with ASD, reported prevalence rates vary considerably across studies, likely reflecting methodological differences and heterogeneity among patient populations [9,10]. These inconsistencies underline the need for further well-designed studies investigating the association between ASD and hearing loss.

Children with ASD may continue to experience persistent pragmatic and social-communication difficulties despite relatively preserved structural language abilities [11]. Heterogeneous developmental trajectories suggest that baseline cognitive and social engagement profiles significantly influence post-implant outcomes [12]. Reduced peer interaction further constrains naturalistic communicative practice [13].

### 1.2. Perspectives on Cochlear Implantation in Children with ASD

Research indicates that children with ASD exhibit reduced neural specialization for speech sounds and diminished social attention to communicative partners, both of which are critical for efficient phonological learning [14]. Furthermore, reduced preference for child-directed speech limits early engagement with linguistically enriched input, thereby constraining vocabulary growth and syntactic development [15].

Language difficulties in ASD extend beyond pragmatic impairments; structural morphosyntactic delays have also been documented, suggesting that both social–cognitive and linguistic processing systems are involved [16]. Neuroimaging studies further reveal atypical development of social brain networks responsible for gaze processing, perspective-taking, and emotional interpretation, which directly influence conversational reciprocity and inferential language use [17].

Additionally, abnormalities in prosody, social attention, and socio-emotional processing may further influence language development and communicative functioning in children with ASD, beyond auditory perception alone [14,15,16,17,18]. Consequently, cochlear implantation in this population requires multidisciplinary management involving audiologists, otolaryngologists, speech therapists, psychologists, and caregivers throughout evaluation and long-term rehabilitation [8,19,20,21,22,23,24]. Family involvement plays a critical role in supporting the developmental, communicative, and educational progress of children with ASD [20].

Cochlear implantation outcomes are shaped by multiple patient- and family-related factors, resulting in substantial variability among recipients [25]. Genetic evaluation may also provide useful prognostic information in children with congenital or hereditary hearing loss, particularly in cases associated with known syndromic or genetic conditions [26,27,28,29].

Hearing loss in children with ASD may present with heterogeneous audiological patterns, including conductive, sensorineural, or mixed forms of impairment [8,30]. In addition, progressive or initially unrecognized hearing loss has been described in some patients, supporting the need for repeated audiological evaluation and long-term hearing surveillance, particularly in children presenting with persistent language delay [8,30].

### 1.3. Impact of ASD on Language Acquisition and Social Interaction

Language disorders in children with autism spectrum disorder are increasingly understood as the result of complex interactions between linguistic impairments and broader social, educational, and environmental factors. Although speech and communication difficulties remain central clinical features of ASD, insufficient consideration of social and cultural context may limit the effectiveness of both diagnostic assessment and therapeutic intervention. Consequently, adapting educational environments and communication strategies to the individual needs of autistic children may play an important role in supporting language development and social participation [20,31].

In children with ASD undergoing cochlear implantation, auditory access represents only one component of language acquisition. Core autism-related mechanisms—including deficits in joint attention, reduced social motivation, impaired Theory of Mind, and pragmatic communication limitations—may moderate the extent to which auditory input is translated into functional language [32].

### 1.4. Core Characteristics of ASD Relevant to Auditory Perception and Communication

Auditory processing abnormalities are increasingly recognized as important features of ASD and may emerge early during neurodevelopment [33]. Children with ASD frequently present difficulties in sound discrimination, auditory filtering, and processing of complex acoustic environments, particularly in the presence of limited verbal abilities [33]. Altered auditory sensitivity and increased susceptibility to sensory overload may further interfere with language development, auditory integration, and social communication [34].

Although the systematic review conducted by Mathew et al. [35] synthesized the available evidence regarding cochlear implantation outcomes in children with Autism Spectrum Disorder (ASD), the literature in this field remains limited, heterogeneous, and continuously evolving. Furthermore, several clinically relevant aspects of cochlear implantation in children with ASD extend beyond the assessment of auditory, speech, and language outcomes alone. The present narrative review aims to complement previous findings by incorporating more recent evidence and by providing a broader clinically oriented perspective on the diagnostic, audiological, rehabilitative, and multidisciplinary challenges encountered throughout the cochlear implantation process in this complex patient population. Particular attention is given not only to communicative, behavioral, developmental, and functional outcomes following cochlear implantation but also to challenges related to audiological assessment, delayed hearing diagnosis, device use and compliance, post-implant mapping considerations, rehabilitation adherence, family-centered care, and long-term multidisciplinary follow-up. In addition, this review emphasizes practical clinical considerations that may support clinical decision-making, expectation setting, rehabilitation planning, and long-term multidisciplinary management for children with ASD undergoing cochlear implantation.

## 2. Materials and Methods

**Figure 1:**
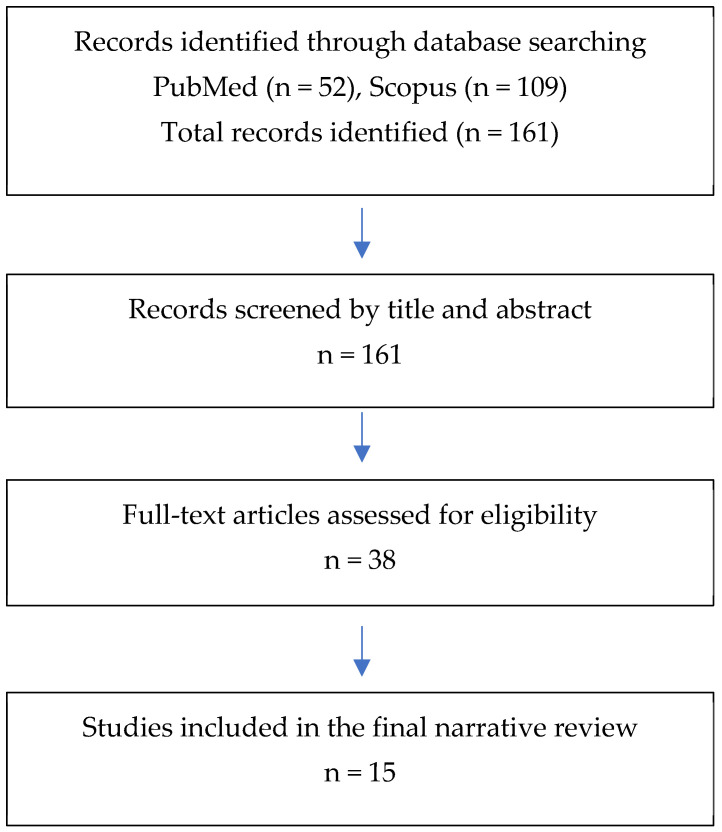
Literature Search and Study Selection Process.

A narrative literature review was conducted to evaluate the current evidence regarding cochlear implantation in children with Autism Spectrum Disorder (ASD), with particular emphasis on audiological assessment, rehabilitation challenges, multidisciplinary care, and post-implant functional outcomes. A structured literature search was performed in PubMed and Scopus to identify articles published in English between January 2000 and January 2026. Additional relevant publications were identified through manual screening of the reference lists of selected articles. The search strategy combined Medical Subject Headings (MeSH) terms and free-text keywords related to cochlear implantation and autism spectrum disorder. The principal Boolean combinations included (“cochlear implant” OR “cochlear implantation”) AND (“autism “OR “ASD” OR “autism spectrum disorder”). The initial PubMed search yielded a manageable number of records and was therefore screened manually without applying additional publication-type filters. In Scopus, conference papers, editorials, notes, books, and book chapters were excluded in order to improve the relevance of the retrieved literature. Studies were considered eligible if they involved pediatric patients (0–18 years) diagnosed with ASD and undergoing cochlear implantation, or if they addressed clinically relevant aspects associated with hearing assessment, auditory rehabilitation, communication development, multidisciplinary management, diagnostic challenges, or post-implant outcomes in this population. Original research articles, observational studies, comparative studies, retrospective reviews, systematic reviews, and narrative reviews published in peer-reviewed journals were included. Articles focusing exclusively on adult populations, non-ASD populations, non-cochlear implant rehabilitation methods, conference abstracts without full-text availability, and non-English publications were excluded. The selection process involved title and abstract screening followed by full-text evaluation of potentially relevant studies (Figure 1).

Particular attention was given to studies addressing audiological assessment challenges, cochlear implant use and compliance, rehabilitation adherence, mapping considerations, communication outcomes, quality of life, and family-centered care. Due to the narrative design of the review and the heterogeneity of the available literature, a qualitative synthesis approach was adopted rather than a formal meta-analysis. A total of 161 records were initially identified (52 from PubMed and 109 from Scopus). Following title and abstract screening, 38 articles underwent full-text assessment. After eligibility assessment and removal of duplicate records, 15 studies were included in the final narrative synthesis (Table 1).

## 3. Results

### 3.1. Diagnostic Challenges of ASD in Children with Profound Hearing Loss

These diagnostic challenges are further illustrated by the findings of Trudeau et al., who evaluated children with severe developmental delay undergoing sedated auditory brainstem response (ABR) testing. In their cohort, 39 children were diagnosed with ASD, most of whom initially presented with concerns related to hearing loss and/or delayed speech development. The authors observed a considerable delay between unsuccessful behavioral audiological assessments and definitive hearing evaluation through ABR, emphasizing the difficulties associated with conventional audiological testing in this patient population [30].

Several studies further emphasized that the coexistence of profound hearing loss and ASD creates important diagnostic challenges because delayed language development, impaired social interaction, and atypical communicative behaviors may overlap between the two conditions [36,37,38]. Consequently, distinguishing communication deficits related to hearing loss from underlying neurodevelopmental impairment may be particularly challenging during early childhood, frequently resulting in delayed ASD recognition and postponed initiation of targeted therapeutic and rehabilitation interventions [30,35,39].

Mancini et al. also reported important limitations of conventional behavioral audiological assessment in children with ASD, since hyperactivity, sensory hypersensitivity, reduced cooperation, and inconsistent responses may interfere with the reliability of hearing evaluation procedures [36]. The authors noted that cochlear implant fitting often requires repeated objective electrophysiological measurements combined with individualized and gradually adjusted programming approaches to support appropriate auditory evaluation and device adaptation [36].

The reviewed studies also highlighted the marked heterogeneity of ASD manifestations among cochlear implant candidates. Variability in sensory processing, communication abilities, behavioral adaptability, and tolerance to auditory stimulation may significantly influence rehabilitation progress, long-term cochlear implant use, and post-implant auditory and language outcomes [35,37,38,39]. Valero et al. reported intermittent cochlear implant use and device rejection in a subset of children with ASD, emphasizing the impact of sensory hypersensitivity and behavioral severity on long-term compliance [38].

**Table 1:** Summary of the included studies.

Author/ Year	Study Design	Sample Size	Population Characteristics	Outcome Domains	Main Findings
**Tavares et al., 2021 [40]**	Systematic review	66 implanted patients	Children with ASD and hearing loss undergoing cochlear implantation	Auditory development, communication, social interaction	Cochlear implantation was associated with improvements in auditory responsiveness and communication.
**Lachowska et al., 2018 [41]**	Retrospective observational study	6 patients	Children with ASD and profound sensorineural hearing loss undergoing cochlear implantation	Communication, auditory responsiveness, parental outcomes	Cochlear implantation improved environmental sound awareness and interaction with caregivers, although oral communication outcomes remained limited.
**Caragli et al., 2023 [5]**	Systematic review	61 included studies	Children with hearing loss and additional disabilities, including ASD	Quality of life, adaptive skills, communication	Cochlear implantation may improve environmental awareness, adaptive functioning, and social interaction in children with additional disabilities.
**Eshraghi et al., 2015 [9]**	Retrospective case–control study	15 ASD patients and 15 controls	Children with ASD undergoing cochlear implantation	Speech perception, speech expression, behavioral outcomes	Most children with ASD demonstrated improvement in speech perception, speech expression, and behavioral responsiveness following cochlear implantation.
**Mancini et al., 2021 [36]**	Multicentre observational study	22 patients	Children with ASD and cochlear implants	Implant fitting, speech perception, language outcomes	ASD severity significantly influenced cochlear implant outcomes and fitting characteristics.
**Mathew et al., 2022 [35]**	Systematic review and meta-analysis	159 patients	Children with ASD undergoing cochlear implantation	Speech perception, communication mode, device use	Cochlear implantation improved speech perception and expression in many patients, although outcomes remained highly variable and poorer than non-ASD patients.
**Trudeau et al., 2021 [30]**	Retrospective chart review	75 children total, 39 with ASD	Children with severe developmental delay, including ASD	Audiological assessment, diagnostic delay	Multiple unsuccessful behavioral audiometry attempts delayed hearing diagnosis and treatment, supporting objective electrophysiological testing.
**Im et al., 2025 [42]**	Retrospective comparative study	10 ASD patients and 20 controls	Cochlear implant recipients with ASD	Speech perception, mapping characteristics	Children with ASD required lower stimulation levels, narrower dynamic ranges, and modified mapping strategies to improve implant tolerance.
**Nasralla et al., 2018 [39]**	Observational questionnaire-based study	14 children, 4 with ASD	Children with multiple disabilities, including ASD, undergoing cochlear implantation	Quality of life, social-emotional development, communication, parental perspective	Cochlear implantation improved social-emotional interaction, environmental awareness, communication abilities, and family quality of life, although oral language outcomes remained variable. Parental expectations required adjustment in children later diagnosed with ASD.
**Jenks et al., 2022 [43]**	Retrospective case review and parent survey	30 children	Children with ASD undergoing cochlear implantation	Speech perception, communication mode, social engagement, educational outcomes	Cochlear implantation improved auditory skills, communication, and social engagement in a proportion of children with ASD, although outcomes remained highly heterogeneous and strongly dependent on consistent device use.
**Datta et al., 2019 [44]**	Retrospective cohort analysis	46 children, 9 with ASD and CI	Deaf children with severe/profound learning difficulties, including ASD	Processor use, communication, cognition, listening skills, rehabilitation outcomes	Cochlear implantation provided functional auditory benefit in most patients, although progress was slow and required persistent long-term rehabilitation support. Additional ASD diagnosis negatively influenced outcomes.
**Donaldson et al., 2004 [45]**	Retrospective review and parental survey	6 children	Children with ASD who underwent cochlear implantation	Speech perception, language development, quality of life, behavioral outcomes	Although language gains were limited, children demonstrated improvement in responsiveness to sound, eye contact, interaction, and overall quality of life following implantation.
**Naderpour et al., 2024 [46]**	Comparative prospective observational study	24 children, 12 with ASD and CI	ASD and non-ASD children undergoing cochlear implantation	Speech intelligibility, auditory performance, language outcomes	Cochlear implantation significantly improved auditory performance in children with ASD; however, speech and language outcomes remained lower compared with non-ASD implanted children. The severity of autism influenced rehabilitation progress.
**Robertson, 2013 [37]**	Retrospective program review	10 children	Children with ASD and severe-to-profound hearing loss undergoing cochlear implantation	Processor use, communication mode, sensory processing, rehabilitation challenges	Children with ASD and cochlear implants presented important communication and sensory-processing challenges, with highly variable outcomes regarding processor use and language development.
**Rodriguez Valero et al., 2016 [38]**	Retrospective case review and survey	22 children	Children subsequently diagnosed with ASD after cochlear implantation	Device compliance, verbal communication, post-implant outcomes	Cochlear implant compliance was highly variable and appeared associated with ASD severity. Most children obtained some degree of auditory or communicative benefit despite inconsistent device use.

### 3.2. The Role of the Multidisciplinary Team in Cochlear Implantation for Children with Autism Spectrum Disorder

Children with autism spectrum disorder (ASD) undergoing cochlear implantation require careful and individualized management that extends beyond the surgical procedure itself and conventional auditory rehabilitation. The reviewed studies showed that children with ASD often present highly variable developmental and cognitive profiles, frequently associated with additional comorbidities that may influence auditory, communicative, and functional outcomes after implantation [5,35,41]. For this reason, several authors emphasized the importance of multidisciplinary assessment and follow-up in order to establish realistic therapeutic objectives, provide appropriate parental counseling, and develop rehabilitation strategies adapted to the specific needs and abilities of each child [5,41].

The reviewed literature emphasized that traditional outcome measures, particularly speech perception and expressive language development, may not fully reflect the overall benefits of cochlear implantation in children with ASD [5,41]. Although not all children develop functional oral communication following implantation, many show improvements in environmental sound awareness, non-verbal communication, adaptive behavior, social interaction, eye contact, responsiveness to environmental stimuli, and family relationships [5,35,40,41]. Auditory pathway development appears highly dependent on early sensory stimulation [25].

Preoperative evaluation plays an important role in the management of children with ASD considered for cochlear implantation. Several authors observed that the diagnosis of ASD is frequently established only after implantation, as early autistic features may resemble the communication difficulties typically associated with profound hearing loss [5,40,41]. For this reason, developmental and neuropsychiatric assessment before and after implantation may help improve diagnostic clarification and guide rehabilitation planning [41]. Ongoing multidisciplinary follow-up is also important for distinguishing limited auditory performance from communication difficulties related to ASD, allowing clinicians to interpret post-implant outcomes within the child’s overall neurodevelopmental profile [5].

Effective multidisciplinary care depends not only on parallel interventions but also on continuous reciprocal information exchange between specialists involved in the rehabilitation process [5,39]. These findings underscore the importance of integrating speech therapy, behavioral intervention, and psychosocial support into long-term rehabilitation programs, while adopting realistic and individualized therapeutic goals focused on functional communication and quality-of-life improvement rather than linguistic milestones alone [39]. Datta et al. also emphasized the importance of continuous parental involvement, realistic expectation counseling, and individualized rehabilitation strategies. The authors reported that progress often developed slowly and required sustained adult support, persistence, and the use of augmentative or alternative communication approaches, particularly in children with ASD and severe additional disabilities [44].

### 3.3. Audiological and Rehabilitation Challenges in Children with ASD Undergoing Cochlear Implantation

Several clinically important challenges have been reported during audiological assessment and cochlear implant candidacy evaluation in children with ASD. Behavioral variability, impaired attention, and inconsistent responses to auditory stimuli may significantly reduce the reliability of conventional behavioral hearing tests. Trudeau et al. reported that repeated behavioral audiometry attempts were frequently unsuccessful in children with severe developmental disorders, often resulting in delayed diagnosis and the need for sedated auditory brainstem response (ABR) testing in order to establish reliable auditory thresholds [30]. Similarly, Mancini et al. emphasized that the coexistence of profound hearing loss and ASD may complicate both hearing evaluation and cochlear implant fitting procedures because communication deficits, behavioral abnormalities, and sensory processing disturbances may interfere with behavioral audiological testing and interpretation of auditory responses [36].

Several studies also reported challenges related to long-term cochlear implant use and rehabilitation adherence in children with ASD. Valero et al. and Robertson described intermittent processor use, inconsistent daily wear, and occasional device rejection, particularly in children presenting with more severe sensory processing abnormalities and behavioral difficulties [35,37]. Auditory hypersensitivity, discomfort related to external device components, behavioral rigidity, and reduced adaptability were identified as important factors that may negatively affect cochlear implant compliance and participation in long-term rehabilitation programs [35,36,37].

Datta et al. emphasized that long-term rehabilitation in children with ASD and severe additional disabilities frequently depended on continuous adult support, prolonged therapeutic input, and the use of augmentative or alternative communication approaches [44].

Post-implant audiological programming and mapping also represent significant areas of complexity in children with ASD undergoing cochlear implantation. Mancini et al. reported difficulties obtaining reliable behavioral feedback during fitting sessions, particularly when determining comfortable stimulation thresholds and optimal electrical charge requirements [36]. In addition, children with ASD may present increased sensitivity to auditory stimulation and reduced tolerance to sound intensity changes during cochlear implant programming sessions. Im et al. reported that these patients often required lower threshold and comfortable stimulation levels, narrower dynamic ranges, and reduced sensitivity and volume settings in order to improve sound tolerance and minimize auditory overstimulation [42]. The authors also emphasized that cochlear implant mapping in children with ASD frequently requires gradual programming adjustments, repeated follow-up sessions, and prolonged adaptation over time [42].

### 3.4. Outcomes of Cochlear Implantation in Children with Additional Disabilities

A systematic review including 7 studies with ASD and hearing loss reported that not all children developed oral communication; however, many demonstrated meaningful gains in social interaction, eye contact, environmental sound awareness, and family engagement following implantation. Among children without additional disabilities, 40% developed fluent oral communication, whereas outcomes were more heterogeneous in those with additional impairments [40].

Children with ASD undergoing cochlear implantation may demonstrate improvements in auditory awareness, responsiveness to environmental sounds, vocalization, and communication abilities; however, speech and language outcomes remain considerably more heterogeneous than in children without additional disabilities [44,45,46]. Several studies reported that communicative progress is often slower and may be significantly influenced by ASD severity, cognitive functioning, sensory processing abnormalities, and rehabilitation adherence. Naderpour et al. demonstrated significant postoperative improvements in auditory performance among children with ASD, although speech intelligibility and language development remained lower compared with non-ASD cochlear implant users [44,46]. Similarly, Mancini et al. reported substantial variability in Categories of Auditory Perception (CAP) and Categories of Language (CL) scores, with more severe ASD manifestations being associated with poorer long-term auditory and language outcomes [36].

Conventional speech and language outcomes may not adequately capture the broader functional benefits of cochlear implantation in children with ASD and additional disabilities. Although oral communication did not develop in all patients, many experienced improvements in social engagement, eye contact, awareness of environmental sounds, and interaction with family members following implantation [41]. Similarly, Eshraghi et al. observed improvements in name recognition, response to verbal requests, attention to environmental sounds, and behavioral interaction after cochlear implantation in children with ASD [9]. Donaldson et al. additionally reported gains in responsiveness to sound, interest in music, vocalization, and quality of life, even in children with limited spoken language development [45].

Despite measurable auditory benefit after cochlear implantation, several studies reported that many children with ASD continue to depend primarily on augmentative or alternative communication methods rather than spoken language alone [35,44]. Datta et al. described the frequent use of combined communication strategies and noted that communicative progress was often slow, requiring prolonged rehabilitation and continuous caregiver support [44]. Similarly, Mathew et al. highlighted the considerable variability in communication outcomes and long-term cochlear implant use, with a large proportion of children remaining nonoral communicators despite functional auditory improvement [35].

## 4. Discussion

The increasing use of cochlear implantation over the past decades, together with the rising recognition of Autism Spectrum Disorder (ASD), has led to growing clinical interest in children presenting with both conditions [1,9]. Although early intervention is generally associated with more favorable language and developmental outcomes [47], the coexistence of ASD and hearing loss continues to pose important diagnostic and rehabilitative challenges [9,48,49]. Delayed language development, reduced social responsiveness, and atypical communicative behaviors may reflect either auditory deprivation or underlying neurodevelopmental impairment, making early differentiation particularly difficult.

Hyperactivity, sensory hypersensitivity, reduced cooperation, and inconsistent responses to auditory stimuli may interfere with the reliability of behavioral hearing evaluation in children with ASD, often requiring repeated objective electrophysiological testing and prolonged diagnostic follow-up [30,36]. These challenges underline the importance of comprehensive developmental assessment and multidisciplinary evaluation in children presenting with persistent language delay, atypical communication patterns, or suspected hearing impairment.

Although current evidence does not clearly demonstrate an increased prevalence of peripheral hearing loss in children with ASD, complete audiological evaluation remains essential whenever ASD is suspected in order to avoid delayed recognition of coexisting hearing impairment [10]. Cochlear implant rehabilitation in children with ASD often requires substantial adaptation of conventional audiological protocols. Behavioral variability, auditory hypersensitivity, intolerance to external device components, and reduced adaptability may negatively affect device compliance and long-term rehabilitation adherence. In addition, post-implant mapping frequently requires gradual programming modifications, lower stimulation levels, and repeated follow-up sessions in order to improve sound tolerance and reduce auditory overstimulation [35,36,37,42].

The findings of this review suggest that outcomes following cochlear implantation in children with ASD should be interpreted within a broader functional and neurodevelopmental framework rather than based exclusively on speech and language measures. Although oral communication may remain limited in some patients, many children demonstrate meaningful improvements in environmental sound awareness, social interaction, adaptive behavior, and responsiveness to auditory stimuli. These observations indicate that conventional cochlear implant outcome measures may not fully reflect the functional benefits observed in this population. The considerable variability in post-implant outcomes likely reflects differences in sensory processing, behavioral characteristics, cognitive functioning, and long-term participation in rehabilitation programs.

### Practical Clinical Recommendations

Children with ASD undergoing cochlear implantation require individualized audiological evaluation, flexible rehabilitation strategies, and long-term multidisciplinary follow-up. Because behavioral audiological assessment may be unreliable in this population, repeated objective electrophysiological testing and gradual cochlear implant programming adjustments are often necessary to optimize auditory evaluation and device tolerance [30,36,42]. Preoperative developmental and neuropsychiatric assessment may also facilitate earlier recognition of ASD-related communication difficulties and support more appropriate rehabilitation planning [5,40,41].

Several limitations of this review should be acknowledged. First, given the narrative design of the study, no formal risk-of-bias assessment or standardized quality appraisal of the included studies was performed. Consequently, the findings should be interpreted with caution, particularly in view of the methodological heterogeneity, small sample sizes, and predominantly retrospective design of the available literature.

Practical clinical considerations derived from the reviewed literature include:

- the use of objective audiological evaluation methods in situations where behavioral testing is unreliable or difficult to perform [30,36,50];
- individualized cochlear implant mapping with gradual programming adjustments and careful adaptation of stimulation levels according to the child’s auditory tolerance [36,42];
- realistic counseling centered on functional communication abilities, daily adaptive functioning, and overall quality-of-life improvement rather than spoken language development alone [5,40,41];
- close long-term collaboration between audiologists, speech and language therapists, psychologists, behavioral specialists, and caregivers throughout the rehabilitation process [5,39,43];
- ongoing caregiver involvement and long-term rehabilitation support, particularly in children who require augmentative or alternative communication methods [35,44].

## 5. Conclusions

In conclusion, cochlear implantation may provide meaningful functional benefits in children with Autism Spectrum Disorder, even when expressive language development remains limited or highly variable. Consequently, post-implant outcomes in children with ASD should not be evaluated exclusively through spoken language development. In many cases, improvements in environmental sound awareness, social interaction, daily communication, and quality of life may represent clinically meaningful benefits even when oral language remains limited. Ongoing caregiver involvement is also important throughout rehabilitation, as family support may influence device use, participation in therapy, and long-term adaptation. These observations further underline the importance of realistic preoperative counseling and individualized therapeutic goals.

Cochlear implantation in children with ASD requires an individualized and multidisciplinary management approach that extends beyond the surgical intervention itself. Comprehensive developmental assessment, realistic preoperative counseling, individualized cochlear implant mapping, and rehabilitation strategies adapted to each child’s communicative and behavioral profile are important components of long-term clinical management. Because sensory hypersensitivity, behavioral variability, and neurodevelopmental characteristics may significantly influence device use and rehabilitation progress, close collaboration between audiologists, otolaryngologists, speech and language therapists, psychologists, behavioral specialists, and caregivers remains essential throughout both the preoperative evaluation and long-term follow-up process.

Overall, ASD should not be considered a contraindication to cochlear implantation, but rather a condition requiring individualized rehabilitation strategies, careful long-term follow-up, and realistic interpretation of outcomes. As cochlear implantation becomes increasingly common in pediatric patients with additional neurodevelopmental comorbidities such as ASD, future research should focus on the development of more standardized and ASD-specific outcome assessment tools capable of better capturing clinically meaningful functional outcomes in children with ASD. Additional prospective studies are also needed to investigate preoperative predictors of cochlear implant outcomes in this patient population, as well as the potential role of systematic auditory screening and earlier audiological assessment in children presenting with neurodevelopmental disorders and communication delay.

## Data Availability

No new data were created or analyzed in this study. Data sharing is not applicable to this article.

## Abbreviations

The following abbreviations are used in this manuscript:

CI	Cochlear Implantation
ASD	Autism Spectrum Disorder
D/HH	Deaf and Hard-of-Hearing
ABR	Auditory Brainstem Response
SNHL	Sensorineural Hearing Loss
ADs	Additional Disabilities
